# EVI1 expression in early-stage breast cancer patients treated with neoadjuvant chemotherapy

**DOI:** 10.1186/s12885-022-10109-1

**Published:** 2022-10-05

**Authors:** Jonas Leichsenring, Valentina Vladimirova, Christine Solbach, Thomas Karn, Beyhan Ataseven, Bruno Valentin Sinn, Jana Barinoff, Volkmar Müller, Jens-Uwe Blohmer, Christian Schem, Knut Engels, Frederik Marmé, Annette Fisseler-Eckhoff, Peter A. Fasching, Elmar Stickeler, Marion van Mackelenbergh, Carsten Denkert, Albrecht Stenzinger, Sibylle Loibl, Stefan Gröschel

**Affiliations:** 1 Pathologisches Institut, Universitätsklinikum Heidelberg, Heidelberg, Germany; 2 German Breast Group (GBG), Neu-Isenburg, Germany; 3 Klinik Für Frauenheilkunde Und Geburtshilfe, Universitätsklinikum, Frankfurt, Germany; 4 Goethe University Hospital Frankfurt, Frankfurt, Germany; 5 Kliniken Essen-Mitte, Essen, Germany; 6 Klinik Für Frauenheilkunde Und Geburtshilfe, Universitätsklinikum München (LMU), München, Germany; 7 Charité – Universitätsmedizin Berlin, corporate member of Freie Universität Berlin and Humboldt Universität Zu Berlin, Department of Pathology, Berlin, Germany; 8 Sankt Gertrauden-Krankenhaus, Berlin, Germany; 9 Universitätsklinikum Hamburg-Eppendorf, Hamburg, Germany; 10 Gynäkologie Mit Brustzentrum, Charité-Universitätsmedizin Berlin, Berlin, Germany; 11 Mammazentrum Hamburg, Hamburg, Germany; 12 Center for Pathology, Cytology and Molecular Pathology, Neuss, Germany; 13 Medizinische Fakultät Mannheim, Universität Heidelberg, Universitätsfrauenklinik Mannheim, Mannheim, Germany; 14 Institut für Pathologie und Zytologie, Helios Dr. Horst Schmidt Kliniken Wiesbaden, Wiesbaden, Germany; 15 University Hospital Erlangen, Erlangen, Germany; 16 Klinik Für Gynäkologie Und Geburtsmedizin, Uniklinik Aachen, Aachen, Germany; 17 Universitätsklinikum Schleswig-Holstein, Klinik für Gynäkologie und Geburtshilfe, Schleswig-Holstein, Kiel, Germany; 18 Institut für Pathologie, Philipps Universität Marburg, Universitätsklinikum Marburg (UKGM), Marburg, Germany; 19 Deutsches Krebsforschungszentrum (DKFZ), Molekulare Leukämogenese A380, 69120 Heidelberg, Germany; 20 Oncology Center Worms, Worms, Germany

**Keywords:** Breast cancer, EVI1, Neoadjuvant chemotherapy

## Abstract

**Background:**

Overexpression of the EVI1 (ecotropic viral integration site 1) oncogene has recently been implicated as a prognostic factor in breast cancer (BC), particularly in triple-negative BC (TNBC). In this study we aimed to investigate frequency and clinical relevance of EVI1 expression in newly diagnosed BC treated with neoadjuvant chemotherapy.

**Methods:**

EVI1 expression was determined by immunohistochemistry using H-score as a cumulative measurement of protein expression in pretherapeutic biopsies of BC patients treated with anthracycline/taxane based neoadjuvant chemotherapy within the GeparTrio trial. EVI1 was analyzed as a continuous variable and dichotomized into low or high based on median expression. Endpoints were pathological complete response (pCR), disease-free survival (DFS) and overall survival (OS).

**Results:**

Of the 993 tumors analyzed, 882 had available subtype information: 50.8% were HR + /HER2-, 15% HR + /HER2 + , 9.8% HR-/HER2 + , and 24.5% TNBC. Median EVI1 H-score was 112.16 (range 0.5–291.4). High EVI1 expression was significantly associated with smaller tumor size (*p* = 0.002) but not with BC subtype. Elevated EVI1 levels were not significantly associated with therapy response and survival in the entire cohort or within BC subtypes. However, TNBC patients with high EVI1 showed a trend towards increased pCR rates compared to low group (37.7% vs 27.5%, *p* = 0.114; odds ratio 1.60 (95%CI 0.90–2.85, *p* = 0.110) and numerically better DFS (HR = 0.77 [95%CI 0.48–1.23], log-rank *p* = 0.271) and OS (HR = 0.76 [95% 0.44–1.31], log-rank *p* = 0.314) without reaching statistical significance.

**Conclusion:**

EVI1 was not associated with response to neoadjuvant therapy or patient survival in the overall cohort. Further analyses are needed to verify our findings especially in the pathological work-up of early-stage HER2-negative BC patients.

**Trial registration:**

NCT00544765.

**Supplementary Information:**

The online version contains supplementary material available at 10.1186/s12885-022-10109-1.

## Background

Although the clinical and genetic heterogeneity of breast cancer has been increasingly recognized in the recent past, and 5-year survival rates have increased to approximately 90% across all stages due to multidisciplinary management and adjuvant therapies of patients, disease recurrence and metastasis still pose a serious challenge for a significant subset of patients. Besides clinically established predictive factors, such as tumor grade and size, hormone receptor (HR) status, lymph node involvement, human epidermal growth factor receptor 2 (HER2) status, and gene expression profiles, novel clinically relevant predictive and prognostic factors are needed to help inform personalization of adjuvant therapies and risk stratification of breast cancer patients. Recently, tumor cell expression status of the EVI1 oncogene has been suggested to serve as a potential biomarker for high-risk breast cancer patients [1].

EVI1 (ecotropic viral integration site 1, also MECOM) is a stem cell regulator in hematopoiesis and a potent oncogene when aberrantly upregulated (EVI1 +) as observed in acute myeloid leukemia and myelodysplastic syndromes [2, 3]. EVI1-deletion in mice is lethal during embryogenesis owing to hematopoietic insufficiency and disturbance of mesenchymal und ectodermal organ development (heart, soft tissues, neural and urogenital tissues, oocytes) [4–7]. Mutations of the coding region of EVI1 are rarely found in cancer, however, EVI1 upregulation or lack of transcriptional silencing during cell differentiation are considered a crucial oncogenic event [8, 9]. *EVI1* maps to chromosome 3q26.2 (*MECOM* = *MDS1 and EVI1 complex locus*) and encodes two major isoforms: the shorter oncogenic EVI1 protein and the longer MDS1-EVI1 protein, which is speculated to function as tumor suppressor. Skewed expression of both isoforms in favor of EVI1 is considered the main oncogenic event [10, 11]. The mechanism of aberrant EVI1 upregulation is frequently unknown, while in myeloid neoplasms it occurs as a consequence of chromosomal 3q26.2 rearrangements [8, 9].

Furthermore, EVI1 + has been implicated in carcinogenesis, particularly in ovarian [12] and breast carcinoma [1]. However, the prognostic importance of EVI1 overexpression in breast cancer remains unclear due to low sample sizes of reported outcomes and lack of patient data from controlled clinical trials. One large, retrospective study analyzed the gene expression level of EVI1 with regard to clinical outcome in patients who had not received systemic therapy. The authors found elevated EVI1 expression in basal subtypes and an association with HR-negativity and poor outcome [13]. Two more recent study explored EVI1 expression in breast cancer by immunohistochemistry. One study of 608 randomly collected breast cancer cases found EVI1 to be expressed in estrogen receptor (ER)-positive as well as ER-negative patients and also found EVI1 to be a prognostic marker in ER-negative and especially in triple-negative breast cancer (TNBC) patients [1]. The second study of 88 TNBC patients also found an association with decreased overall survival (OS) and disease-free survival (DFS) [14]. No information was given regarding the nature of the tissue analyzed or the treatment received.

Our aim was to elucidate the prevalence and prognostic impact of EVI1 deregulation on breast cancer subtypes in a well-defined clinical trial cohort.

## Materials and methods

### Patients

The neoadjuvant GeparTrio pilot [15] and main (NCT00544765) [16] trials were prospective phase II and III trials including 2,357 patients with breast cancer (cT2-4 cN0-3 cM0) recruited between 2001 and 2005. Patients received two cycles of docetaxel, adriamycin, and cyclophosphamide (TAC), and response was evaluated by ultrasound. Responders received four more cycles of TAC (pilot study) or were randomly assigned to four or six cycles of TAC (main study). Non-responders were randomized to receive either four cycles of TAC or four cycles of vinorelbine and capecitabine. HR-positivity was defined as ≥ 10% of tumor cells with ER and/or progesterone receptor (PgR) expression. Human epidermal growth factor receptor 2 (HER2) positivity was determined by immunohistochemistry (HER2 score 3 +) and in situ hybridization where appropriate (HER2/CEP17 ratio > 2.2). Endocrine treatment for five years was planned for patients with HR-positive disease but was not part of the protocol. HER2 therapy was not available at that time. We used all samples with available material in the central biobank. The trials were approved by the relevant ethics committees and competent authorities. All patients provided written informed consent for study participation, biomaterial, and data collection.

Breast cancer subtypes were defined as follows: HR + /HER2- (ER-positive and/or PgR-positive, HER2-negative), HR + /HER2 + (ER-positive and/or PgR-positive, HER2-positive), HR-/HER2 + (ER-negative and PgR-negative, HER2-positive), and TNBC (ER-negative, PgR-negative, HER2-negative).

### Immunohistochemistry

Formalin-fixed paraffin embedded tissue material was retrieved from the archives at the German Breast Group, Germany, where tissue microarrays (TMA) were prepared. Immunohistochemistry was performed on 0.5 µm tissue sections on TOMO slides (TOM-1190, Engelbrecht), which were dried at 37 °C overnight at the Institute of Pathology at the University Hospital Heidelberg, Germany. Slides were stained with EVI1 antibody (Cat. No. 2593, Clone C50E12, Cell Signaling) at a dilution of 1:250 using a Ventana Benchmark Ultra following standard protocol.

Slides of the EVI1-stained TMA were scanned at 40 × magnification using a Hamamatsu Slide Scanner (Hamamatsu Photonics K.K.) and saved as NanoZoomer Digital pathology Image (ndpi) format after visual inspection of staining quality under a microscope by an expert pathologist. Digital images were loaded into QuPath (version 0.1.2) and a grid was laid over the images using the TMA dearrayer function. The grid was manually adjusted to encompass all cores. Color deconvolution was performed using the estimate stain vector function [17] and auto detection (minimal channel OD 0.05, max total OD 1, Ignore extrema 1%). Cells were detected by the watershed cell detection function based on the optical density sum and standard parameters. A cell classifier was built using the detection classifier function based on a random trees model with individual annotation of over 24,000 tumor, stromal and immune cells as well as areas of necrosis. Automated cell classification was visually inspected by expert pathologists for each TMA.

EVI1 expression levels were determined using H-score (https://www.ascopost.com/issues/april-10-2015/calculating-h-score) defined as a cumulative measurement of staining intensity and percentage of positive cells. EVI1 protein expression was analyzed as a continuous and dichotomized variable. EVI1 score was categorized based on median cut-off into low (≤ 112.16 score) or high (≥ 112.17 score).

### Statistical methods and analysis

The endpoints included pathological complete response (pCR), DFS, and OS. pCR was defined as no residual invasive and no noninvasive disease in any excised breast or regional node tissue [16]. DFS and OS were defined as the time (in months) from random assignment to the event; patients without event were censored at the time of the last contact [18, 19]. Events for DFS were any loco-regional (ipsilateral breast or local/regional lymph nodes) recurrence of disease, any contralateral breast cancer, any distant recurrence of disease, any secondary malignancy, or death as a result of any cause, whichever occurred first. OS was defined as the time since random assignment until death as a result of any cause [18].

Associations between EVI1 expression and clinico-pathological parameters were assessed by Fisher’s exact test (binary parameters), Chi-square test (categorical parameters with more than two categories), and Mann–Whitney (continuous parameters) test. The distribution of EVI1 score among breast cancer subtypes was assessed using Kruskal–Wallis test. Survival was analyzed by Kaplan–Meier product-limit method and compared between groups using the log-rank test. Median-follow-up time was estimated with the inverse Kaplan–Meier method. Logistic regression and Cox proportional hazard models with 95% confidence interval (CI) were used to correlate EVI1 expression with pCR and survival (DFS and OS), respectively, overall and in subgroups. For regression analyses EVI1 continuous expression was transformed at units log10 increase. The independent predictive/prognostic values were evaluated in multivariate regression models including age (> 50 vs ≤ 50 years), tumor size (cT3-4 vs cT1-2), nodal status (cN + vs cN-), tumor grade (G3 vs G1-2), and histology (non-ductal vs ductal). The interaction between subgroups was assessed by bivariate regression models. All reported *p*-values were two-sided, and *p* < 0.05 was considered statistically significant. All statistical analyses were performed using SPSS 25.0 (IBM SPSS Statistics 25).

## Results

### EVI1 expression in breast cancer

**Fig. 1:**
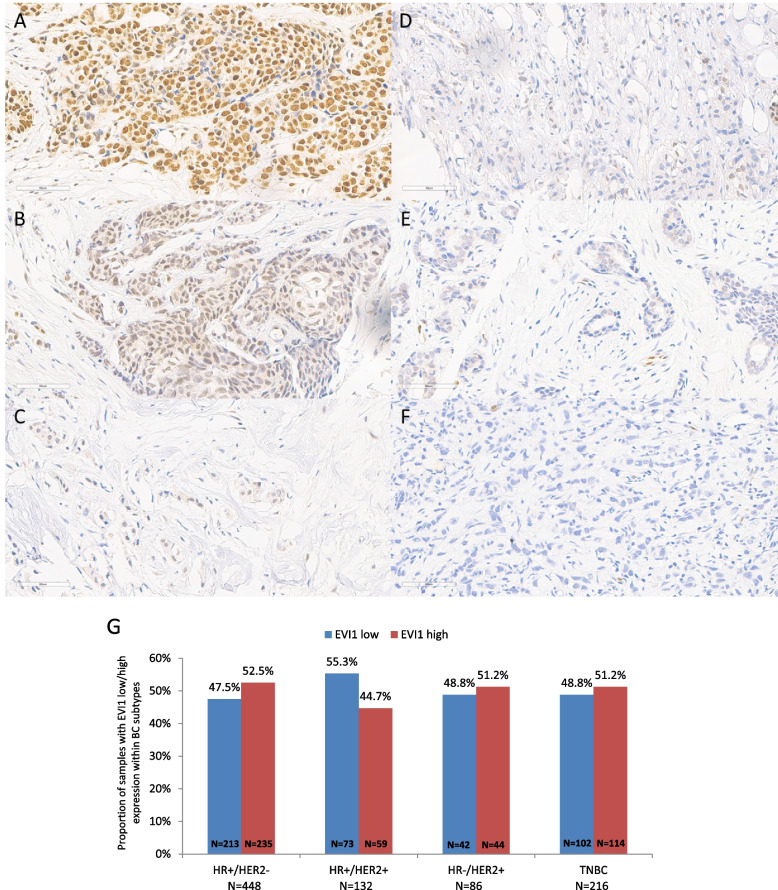
Distribution of EVI1 protein expression in breast cancer: Panels A-F show exemplary immunohistochemical micrographs across a range of EVI1 H-score values (entire cohort *N* = 993): **A** H-score 291 **B** H-score 205, **C** H-score 154, **D** H-score 105, **E** H-score 60 and **F** H-score 3. EVI1 expression levels were determined using H-score defined as a cumulative measurement of staining intensity and percentage of positive cells. **G** Bar chart plots of EVI1 dichotomized expression within breast cancer subtypes. EVI1 H-score was categorized based on the median cut-off into low (≤ 112.16 score) or high (≥ 112.17 score). Note, 882/993 patients with available BC subtype information were included in this analysis. Abbreviations: HR, hormone receptor; HER2, human epidermal growth factor receptor 2; TNBC, triple-negative breast cancer; BC, breast cancer

A total of 993 breast cancer patients with evaluable immunohistochemical EVI1 H-score and available clinical and follow-up data were included in the analysis (Fig. 1A-F; Supplementary Fig. 1). Based on the median EVI1 H-score of 112.16 (range 0.5–291.4) in the entire cohort, EVI1 expression was categorized into low (≤ 112.16 score) or high (≥ 112.17 score). Of the 993 tumors analyzed, 882 had available BC subtype information: 50.8% (*n* = 448) were HR + /HER2-, 15% (*n* = 132) HR + /HER2 + , 9.8% (*n* = 86) HR-/HER2 + , and 24.5% (*n* = 216) TNBC. Across BC subtypes, the distribution of EVI1 was comparable (Fig. 1G, Supplementary Fig. 2).

Median age of the entire patient cohort was 49 (range 23–80) years. Analyzing EVI1 as dichotomized variable based on median cut-off demonstrated that high EVI1 levels compared to the low EVI1 levels were significantly associated with a smaller tumor size (cT1-2, 53.4% vs 46.6%; *p* = 0.002) (Table 1). No significant correlation with other patient and tumor characteristics was observed. Across BC subtypes, high EVI1 expression was significantly associated with smaller tumor size in HR + /HER2- (cT1-2, *p* = 0.004) but not in the other subtypes (Supplementary Table 1).

**Table 1:** Baseline patient and tumor characteristics according to EVI1 expression

Parameter	Category	EVI1-low (≤ 112.16) *N *= 497 N (%)	EVI1-high (≥ 112.17) *N* = 496 N (%)	Overall *N* = 993 N	*p*-value
**Age, years**				993	
	≤ 50	268 (49.7%)	271 (50.3%)	539	0.849
	> 50	229 (50.4%)	225 (49.6%)	454	
	median (range)	49.0 (23–80)	49.0 (24–74)	49.0 (23–80)	0.723
**Tumor size**				990	
	cT1-2	321 (46.6%)	368 (53.4%)	689	0.002
	cT3-4a-d	173 (57.5%)	128 (42.5%)	301	
	missing	3	0	3	
**Nodal status**				976	
	cN-negative	219 (50.3%)	216 (49.7%)	435	0.949
	cN-positive	271 (50.1%)	270 (49.9%)	541	
	missing	7	10	17	
**Tumor grade**				943	
	G1-2	299 (51.7%)	279 (48.3%)	578	0.423
	G3	179 (49.0%)	186 (51.0%)	365	
	missing	19	31	50	
**Histological type**				993	
	Ductal invasive	449 (50.4%)	441 (49.6%)	890	0.468
	Non-ductal	48 (46.6%)	55 (53.4%)	103	
**BC subtype**				882	
	HR + /HER2-	213 (47.5%)	235 (52.5%)	448	0.435
	HR + /HER2 +	73 (55.3%)	59 (44.7%)	132	
	HR-/HER2 +	42 (48.8%)	44 (51.2%)	86	
	TNBC	102 (47.2%)	114 (52.8%)	216	
	missing	67	44	111	
**Stromal TILs (*N* = 523)**	Median (range)	20.0 (0.0–90.0)	20.0 (0.0–100.0)	20.0 (0.0–100.0)	0.848
	missing	253	217	470	
**Ki-67% (*N* = 770)**	Median (range)	28.5 (0.5–99.0)	27.5 (0.0–100.0)	28.0 (0.0–100.0)	0.862
	missing	94	129	223	

*HR* hormone receptor, *HER2* human epidermal growth factor receptor 2, *TNBC* triple-negative breast cancer

### Association of EVI1 expression with therapy response

**Fig. 2:**
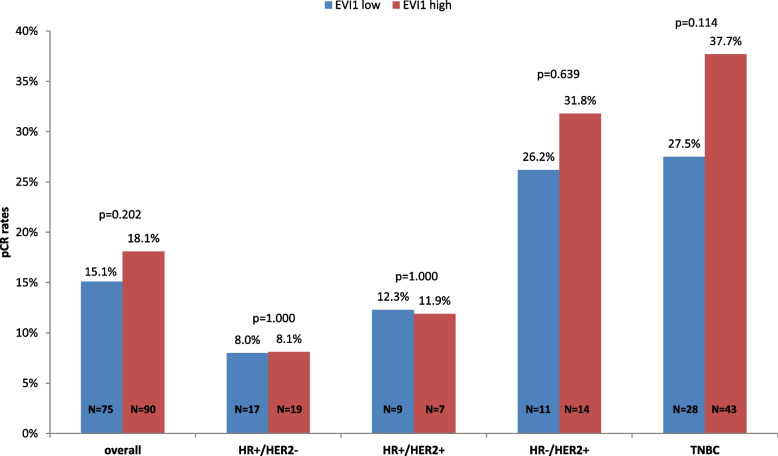
Correlation of EVI1 expression with pCR (ypT0 ypN0). Note, pCR rates in the entire cohort and among breast cancer subtypes were estimated by 2-sided chi-square test. For details, please see Supplementary Table 3. Abbreviations: pCR, pathological complete response; HR, hormone receptor; HER2, human epidermal growth factor receptor 2; TNBC, triple-negative breast cancer; N, number of patients analyzed

Of the 993 patients analyzed, 165 achieved a pCR. Overall, there was no significant correlation of increased EVI1 continuous expression with pCR in univariate model (OR 1.16 [95%CI 0.82–1.65], *p* = 0.404) (Table 2). EVI1 expression also did not predict achievement of a pCR after adjustment for clinical parameters age, tumor size, nodal status, and histological type (OR 1.13 [95%CI 0.77–1.65], *p* = 0.542) (Supplementary Table 2). Across EVI1 groups, 18.1% of patients with high EVI1 expression versus 15.1% of patients with low EVI1 (*p* = 0.202) achieved a pCR (Fig. 2, Supplementary Table 3). Similarly, both univariate and multivariate analysis showed no influence of EVI1 dichotomized expression on therapy response (Table 2, Supplementary Table 2).

**Table 2:** Correlation of EVI1 expression with therapy response and clinical outcomes in the entire cohort

Endpoint	Category	Odds ratio/ Hazard ratio (95% CI)	*p*-value*
**pCR (ypT0 ypN0)**	EVI1 continuous	1.16 (0.82–1.65)	0.404
**DFS**	EVI1 continuous	0.89 (0.70–1.12)	0.305
**OS**	EVI1 continuous	0.81 (0.61–1.07)	0.129
**pCR (ypT0 ypN0)**	EVI1 high vs low	1.25 (0.89–1.74)	0.197
**DFS**	EVI1 high vs low	0.91 (0.71–1.17)	0.460
**OS**	EVI1 high vs low	0.85 (0.62–1.15)	0.293

Note, for regression analyses EVI1 continuous expression was transformed at units log10 increase

*CI* Confidence interval, *pCR* Pathological complete response, *DFS* Disease-free survival *OS* overall survival

^*^ for EVI1 as continuous variable *p*-values according to regression logistic (pCR) and Cox (DFS and OS) analyses, for EVI1 as dichotomized variable *p*-values according to regression logistic (pCR) analysis and log-rank test (DFS and OS)

Subgroup analyses stratified by BC subtype included 882 patients with centrally confirmed ER, PR and HER2 status. No additional predictive information of EVI1 continuous expression on pCR was found (Table 3). In TNBC, patients with high EVI1 expression showed increased pCR rates compared to low group (37.7% vs 27.5%, *p* = 0.114) (Supplementary Table 3) with OR of 1.60 (95%CI 0.90–2.85, *p* = 0.110) (Fig. 2). There was also no significant association with pCR in the other BC subtypes (Table 3). No additional predictive information of EVI1 expression was seen among BC subtypes in multivariate model (Supplementary Table 4).

**Table 3:** Correlation of EVI1 expression with therapy response and clinical outcomes among BC subtypes

BC subtype	EVI1	pCR (ypT0 ypN0)			DFS			OS		
		**Odds ratio** **(95% CI)**	*p*-value*	**Int. *p*-value**	**Hazard ratio** **(95% CI)**	*p*-value*	**Int. *p*-value**	**Hazard ratio** **(95% CI)**	*p*-value*	**Int. *p*-value**
**HR + /HER2-**	continuous	1.18 (0.56–2.50)	0.666	0.555	1.11 (0.73–1.70)	0.617	0.140	1.00 (0.59–1.69)	0.988	0.304
**HR + /HER2 + **	continuous	0.81 (0.32–2.05)	0.662		0.87 (0.52–1.45)	0.581		0.76 (0.40–1.46)	0.411	
**HR-/HER2 + **	continuous	1.52 (0.53–4.41)	0.440		0.81 (0.40–1.65)	0.562		0.62 (0.29–1.34)	0.226	
**TNBC**	continuous	1.41 (0.79–2.52)	0.249		0.75 (0.50–1.13)	0.165		0.74 (0.47–1.18)	0.204	
**HR + /HER2-**	EVI1 high vs low	1.01 (0.51–2.01)	0.968	0.283	1.01 (0.69–1.48)	0.961	0.274	1.00 (0.61–1.64)	0.992	0.290
**HR + /HER2 + **	EVI1 high vs low	0.96 (0.33–2.75)	0.376		0.98 (0.54–1.79)	0.960		0.81 (0.37–1.78)	0.602	
**HR-/HER2 + **	EVI1 high vs low	1.32 (0.52–3.35)	0.566		0.93 (0.46–1.91)	0.849		0.67 (0.27–1.63)	0.370	
**TNBC**	EVI1 high vs low	1.60 (0.90–2.85)	0.110		0.77 (0.48–1.23)	0.271		0.76 (0.44–1.31)	0.314	

Note, for regression analyses EVI1 continuous expression was transformed at units log10 increase

*Int* Interaction, *CI* Confidence interval, *HR* Hormone receptor, *HER2* Human epidermal growth factor receptor 2, *TNBC* Triple-negative breast cancer

^*^ for EVI1 as continuous variable *p*-values according to regression logistic (pCR) and Cox (DFS and OS) analyses, for EVI1 as dichotomized variable according to regression logistic (pCR) analysis and log-rank test (DFS and OS)

### Association of EVI1 expression with survival

After a median follow-up of 74.5 (range 0.0–189.5) months, 278 patients had a relapse and 180 died. EVI1 continuous expression neither impacted DFS (HR = 0.89 [95%CI 0.70–1.12), *p* = 0.305) nor OS (HR = 0.81 [95%CI 0.61–1.07], *p* = 0.129) in the entire cohort (Table 2). In the multivariate model, EVI1 continuous expression did also not add additional prognostic value (Supplementary Table 2). Similarly, dichotomized high EVI1 levels did not correlate with DFS and OS in uni- and multivariate models in the entire cohort (Table 2, Supplementary Table 2).

**Fig. 3:**
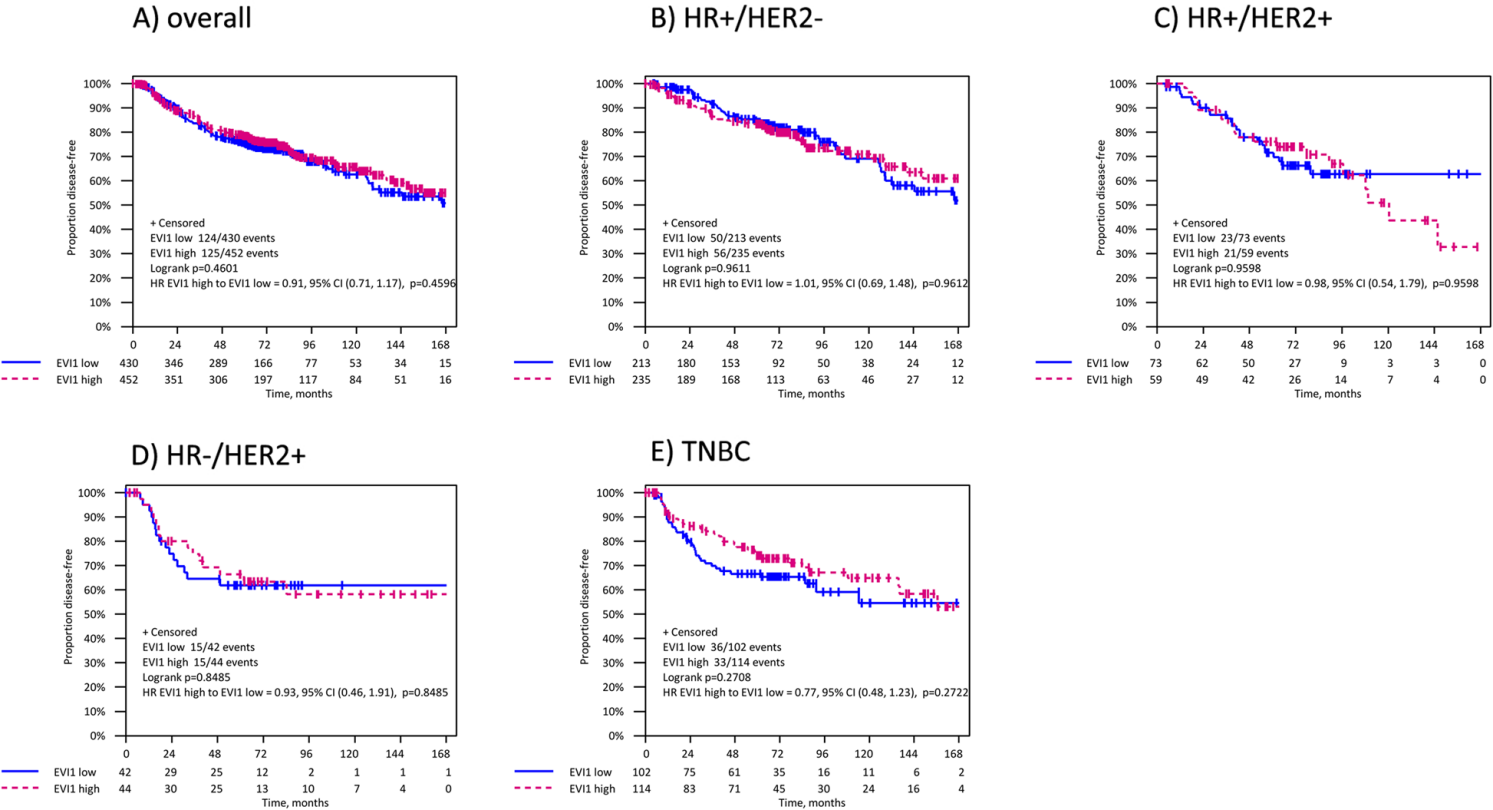
Kaplan–Meier estimates for DFS in the entire cohort and in breast cancer subtypes according to EVI1 expression. Abbreviations: DFS, disease-free survival; HR, hormone receptor; HER2, human epidermal growth factor receptor 2; TNBC, triple-negative breast cancer

**Fig. 4:**
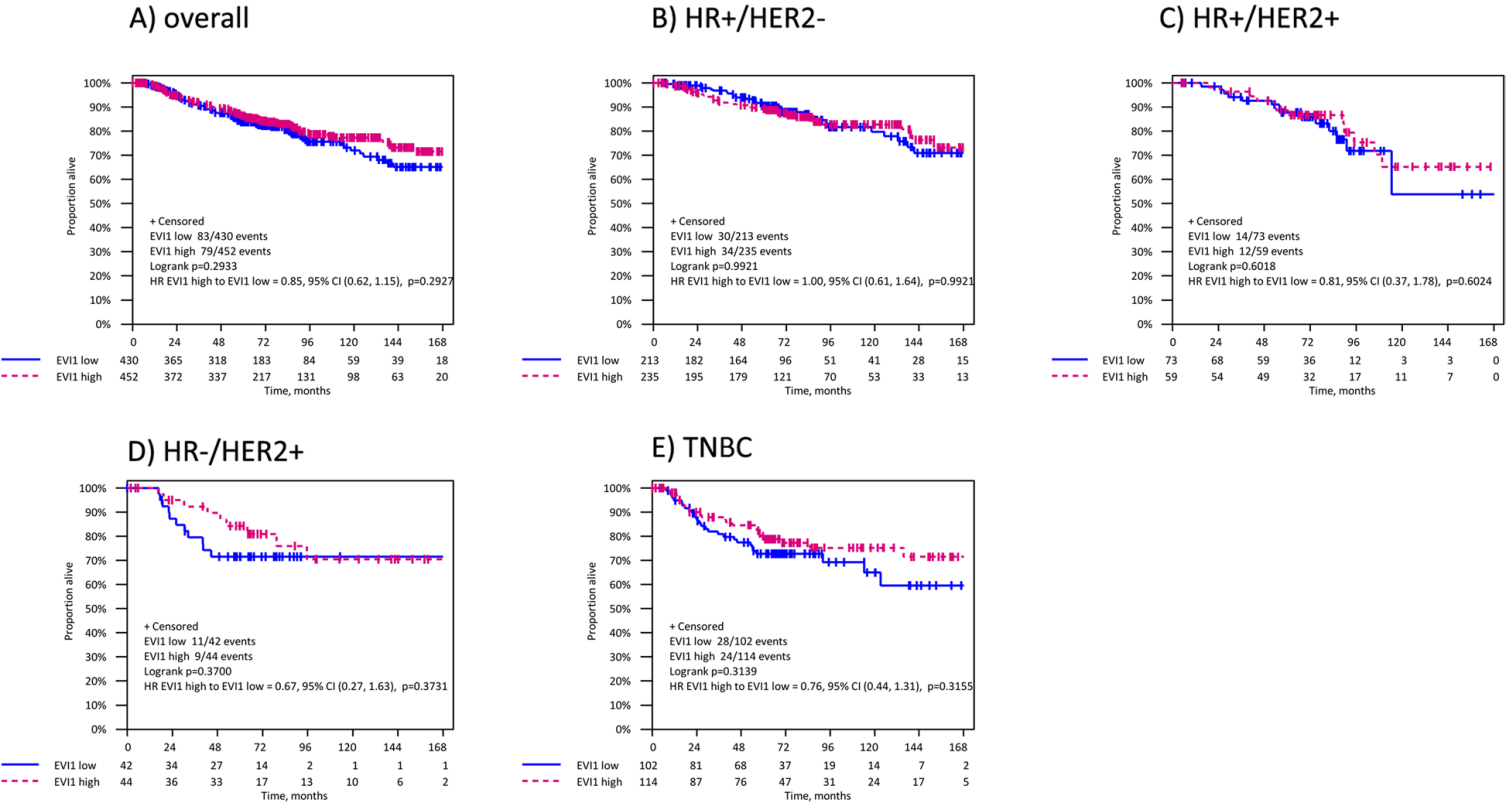
Kaplan–Meier estimates for OS in the entire cohort and in breast cancer subtypes according to EVI1 expression. Abbreviations: OS, overall survival; HR, hormone receptor, HER2, human epidermal growth factor receptor 2; TNBC, triple-negative breast cancer

Across BC subtypes, patients with elevated EVI1 expression showed better but not statistically significant DFS and OS in TNBC (DFS, HR = 0.77 [95%CI 0.48–1.23], *p* = 0.165 and OS, HR = 0.74 [95%CI 0.47–1.18], *p* = 0.204) (Table 3). Multivariate analysis revealed no prognostic relevance of EVI1 continuous score in any of the BC subtypes with regards to DFS and OS (Supplementary Table 4). Comparable results for TNBC patients were observed when analyzing EVI1 dichotomized expression (DFS: HR = 0.77 [95%CI 0.48–1.23], log-rank *p* = 0.271 and OS: HR = 0.76 [95% 0.44–1.31], log-rank *p* = 0.314) (Tables 3 and Supplementary Table 4, Figs. 3 and 4).

Further analysis of the EVI1 prognostic relevance on the 828 patients not achieving pCR showed no significant correlation of high EVI1 expression with DFS (HR = 0.94 [95%CI 0.73–1.22], log-rank *p* = 0.660) or OS (HR = 0.91 [95%CI 0.67–1.26], log-rank *p* = 0.585) in the entire cohort and among BC subtypes (Supplementary Fig. 3, Supplementary Table 5).

## Discussion

This study evaluated the effect of EVI1 protein expression on clinical outcomes in 993 breast cancer patients from the randomized GeparTrio trial. To the best of our knowledge, this is the first study to analyze EVI1 protein expression on breast cancer subtypes as well as the impact of EVI1 expression on response to anthracycline/taxane based neoadjuvant chemotherapy. Our results demonstrated that EVI1 expression in the different breast cancer subtypes did not have any prognostic importance for therapy response and survival.

The molecular regulation and functional relevance of EVI1 expression in breast cancer are largely unexplored. Analysis of a large cohort of primary samples did not detect significant gene rearrangements or copy-number gains in breast cancer, indicating that activation of the EVI1 locus in breast cancer follows different principles than in myeloid leukemia, possibly via miRNA regulation [1, 13].

The rate of TNBC patients with high EVI1 levels (53%) was comparable to 59% reported in a study analyzing a small cohort of only TNBC (*n* = 88) using a different EVI1 staining score system [14]. Interestingly, we found a significant association of high EVI1 expression with smaller tumor size in the entire cohort (*p* = 0.002) and in HR + /HER2- BC (*p* = 0.004) but not in TNBC (*p* = 0.187).

Overall, patients with elevated EVI1 expression demonstrated numerically higher pCR rate compared to low EVI1 group after neoadjuvant chemotherapy. In HR-negative/HER2-positive and TNBC, a high EVI1 expression was associated with increased pCR compared to low group (31.8% vs 26.2% for HR-negative/HER2-positive and 37.7% vs 27.5% for TNBC) but these associations were not statistically significant. A comprehensive explanation as to why high EVI1 levels, previously reported as an adverse prognostic factor, associated with better pCR rate in this study remains elusive. A possible interpretation of this somewhat contradictory finding may be that our EVI1 IHC assay identified more patients with highly proliferative tumors being more responsive to cytotoxic regimens rather than chemotherapy resistant clinical phenotypes as for example in myeloid leukemias. At any rate, higher EVI1 expression did not add any additional predictive value to the neoadjuvant therapy response in this cohort.

With regards to EVI1 prognostic impact on survival, EVI1 protein expression was not associated with improved DFS and OS in the entire cohort or in any breast cancer subtype. However, TNBC patients with elevated EVI1 expression had better DFS and OS. In the study of Wang et al., EVI1 was identified as a prognostic marker of OS in ER-negative breast cancer and especially TNBC in univariate analysis. This prognostic effect was not detected in ER-positive breast cancer or the entire cohort and was lost when ER-/HER2 + breast cancer subgroup was separately analyzed [1].

The major strength of our analysis was the large sample size of 993 patients that allowed for the estimation of EVI1 expression and clinical relevance in different breast cancer subtypes. Patients with primary breast cancer and centrally confirmed ER, PgR, and HER2 status were enrolled in the randomized prospectively conducted phase III GeparTrio trial. All patients included in our analysis received comparable anthracycline/taxane-based neoadjuvant chemotherapy. Moreover, this report is the first to examine the impact of EVI1 expression on response in breast cancer patients treated with neoadjuvant chemotherapy.

A limitation of the study was the smaller number of events observed in all breast cancer subtypes, limiting the power of the multivariate analyses. Furthermore, methodical differences in EVI1 expression analyses may account for the contradicting results on the EVI1 prognostic relevance as found here. EVI1 protein immunophenotyping as performed in this study may not fully capture the entirety of patients with EVI1 increased gene expression and thus miss patients at risk. Hence, further prospective validation studies with larger sample sizes and available follow-up data are warranted to validate the prognostic relevance of EVI1 in patients with early-stage BC.

In conclusion, EVI1 was not associated with response to neoadjuvant therapy or patient survival in this cohort of breast cancer patients. Further analyses are needed to verify our findings especially in the pathological work-up of newly-diagnosed BC patients.

## Supplementary Information

**Additional file 1: Supplementary Figure 1.** Flow diagram of patients included in the analysis set from GeparTrio study.

**Additional file 2: Supplementary Figure 2.** Distribution of EVI1 continuous expression in breast cancer: A: Histogram of EVI1 continuous expression in the entire cohort (*N*=993); B: Boxplots of EVI1 continuous expression within breast cancer subtypes. Note, 882/993 patients with available BC subtype information were included in this analysis. Horizontal bold lines present EVI1 median values of 116.65 in HR+/HER2-, 98.67 in HR+/HER2+, 114.77in HR-/HER2+, and 115.64 in TNBC; boxes present the interquartile range between first quartile and third quartile of the dataset; whiskers present the range from the smallest and the highest number of the dataset). Abbreviations: HR, hormone receptor; HER2, human epidermal growth factor receptor 2; TNBC, triple-negative breast cancer.

**Additional file 3: Supplementary Figure 3.** Kaplan-Meier estimates of DFS (A) and OS (B) according to EVI1 expression in the non-pCR subgroup. Abbreviations:pCR, pathological complete response; DFS, disease-free survival; OS, overallsurvival.

**Additional file 4: Supplementary Table 1.** Correlation of baseline patient and tumor characteristics with EVI1 expression among BC subtypes. **Supplementary Table 2.** Multivariate analysis of EVI1 prognostic value in the entire cohort. **Supplementary Table 3. **pCR (ypT0 ypN0) rates overall and among breast cancer subtypes. **Supplementary Table 4.** Multivariate analysis of EVI1 prognostic value across BC subtypes. **Supplementary Table 5.** Univariate analysis of EVI1 prognostic value in patients with residual disease overall and among BC subtypes.

## Data Availability

The data underlying the results presented in the study are available from the corresponding author or German Breast Group on reasonable request. Some restrictions apply due to confidentiality of patient data. Since these data are derived from a prospective clinical trial with ongoing follow up collection there are legal and ethical restrictions to share sensitive patient related data publicly. Interested groups may use the “Cooperation Proposal Form” on https://www.gbg.de/en/research/trafo.php.

## Abbreviations

EVI1: Ecotropic Viral Integration 1 oncogene
TNBC: Triple-negative breast cancer
ER: Estrogen receptor
DFS: Disease-free survival
OS: Overall survival
pCR: Pathological complete response
TAC: Docetaxel, adriamycin, and cyclophosphamide
PgR: Progesterone receptor
HER2: Human epidermal growth factor receptor 2
IHC: Immunohistochemistry
TMA: Tissue microarray
CI: Confidence interval
OR: Odds ratio
