# Supplementary material for: EVI1 expression in early-stage breast cancer patients treated with neoadjuvant chemotherapy
Source: BMC Cancer. 2022 Oct 5;22:1040. doi: 10.1186/s12885-022-10109-1 (PMC9533588; doi:10.1186/s12885-022-10109-1)
Supplement: Supplementary file 1 — Additional file 1: Supplementary Figure 1. Flow diagram of patients included in the analysis set from GeparTrio study. [file 12885_2022_10109_MOESM1_ESM.pptx]

## Slide 1
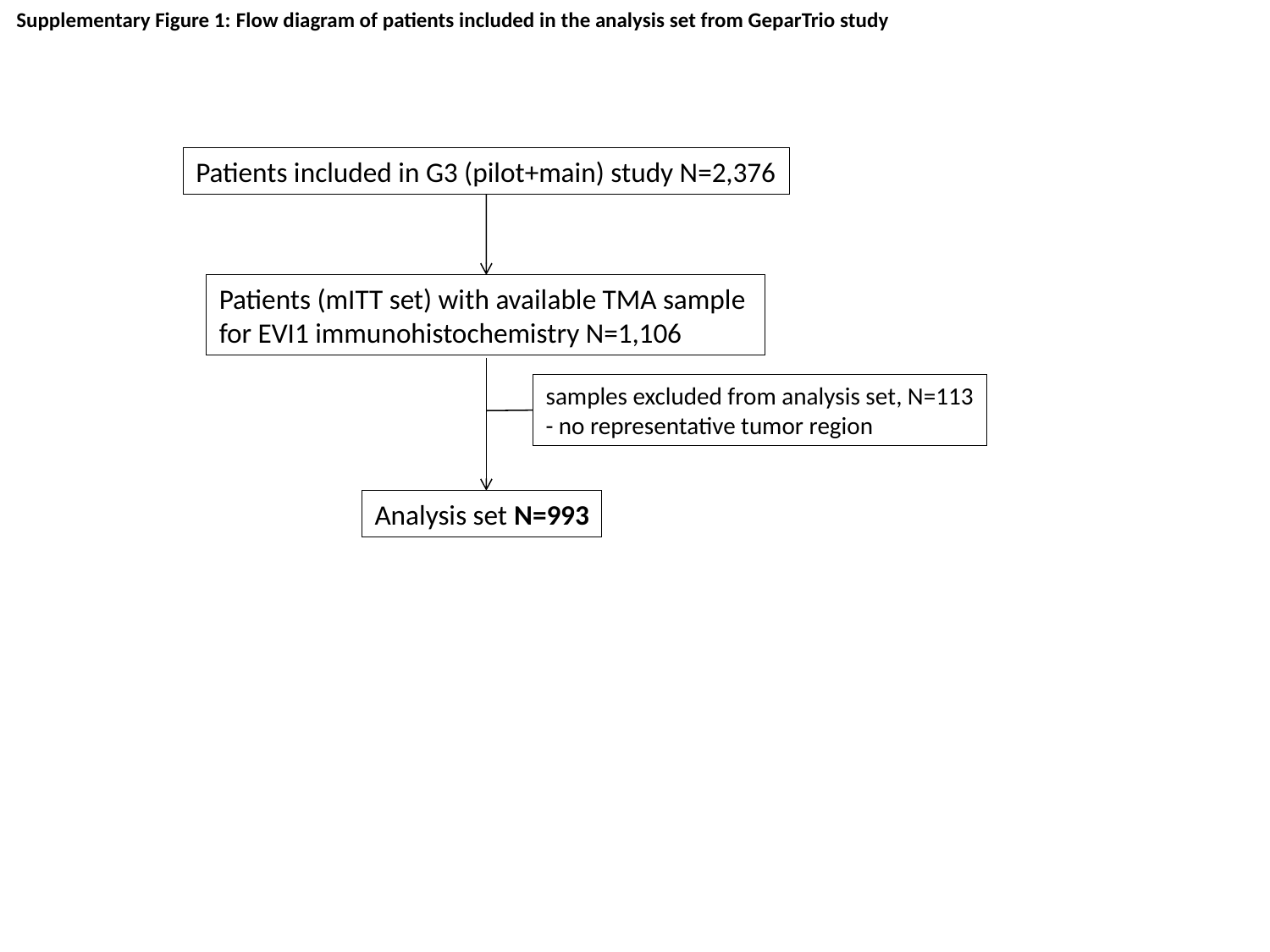

Supplementary Figure 1: Flow diagram of patients included in the analysis set from GeparTrio study
Patients included in G3 (pilot+main) study N=2,376
Patients (mITT set) with available TMA sample
for EVI1 immunohistochemistry N=1,106
samples excluded from analysis set, N=113
- no representative tumor region
Analysis set N=993
