# Supplementary material for: EVI1 expression in early-stage breast cancer patients treated with neoadjuvant chemotherapy
Source: BMC Cancer. 2022 Oct 5;22:1040. doi: 10.1186/s12885-022-10109-1 (PMC9533588; doi:10.1186/s12885-022-10109-1)
Supplement: Supplementary file 2 — Additional file 2: Supplementary Figure 2. Distribution of EVI1 continuous expression in breast cancer: A: Histogram of EVI1 continuous expression in the entire cohort (N=993); B: Boxplots of EVI1 continuous expression within breast cancer subtypes. Note, 882/993 patients with available BC subtype information were included in this analysis. Horizontal bold lines present EVI1 median values of 116.65 in HR+/HER2-, 98.67 in HR+/HER2+, 114.77in HR-/HER2+, and 115.64 in TNBC; boxes present the interquartile range between first quartile and third quartile of the dataset; whiskers present the range from the smallest and the highest number of the dataset). Abbreviations: HR, hormone receptor; HER2, human epidermal growth factor receptor 2; TNBC, triple-negative breast cancer. [file 12885_2022_10109_MOESM2_ESM.pptx]

## Slide 1
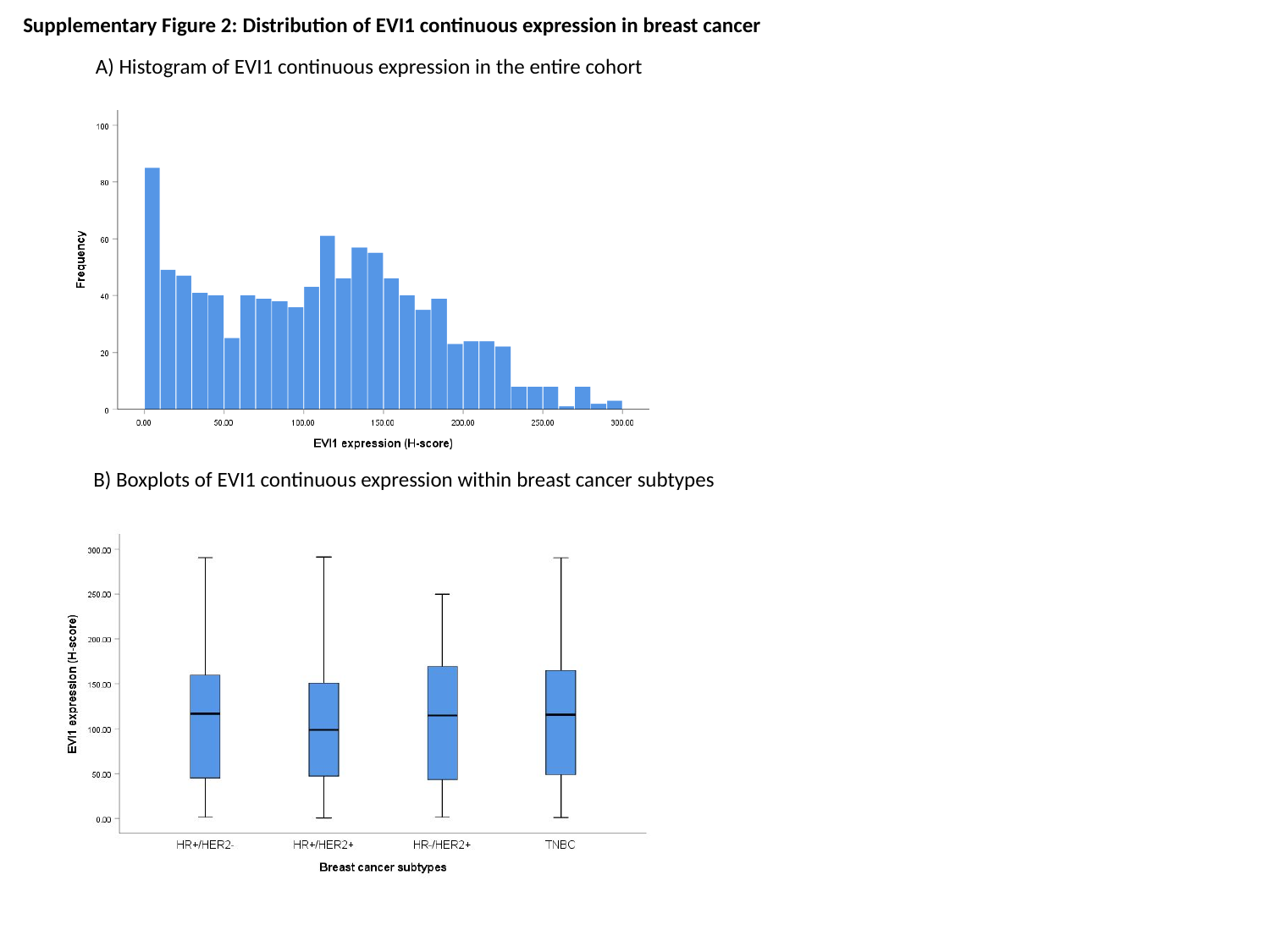

Supplementary Figure 2: Distribution of EVI1 continuous expression in breast cancer
A) Histogram of EVI1 continuous expression in the entire cohort
B) Boxplots of EVI1 continuous expression within breast cancer subtypes
