# Supplementary material for: EVI1 expression in early-stage breast cancer patients treated with neoadjuvant chemotherapy
Source: BMC Cancer. 2022 Oct 5;22:1040. doi: 10.1186/s12885-022-10109-1 (PMC9533588; doi:10.1186/s12885-022-10109-1)
Supplement: Supplementary file 3 — Additional file 3: Supplementary Figure 3. Kaplan-Meier estimates of DFS (A) and OS (B) according to EVI1 expression in the non-pCR subgroup. Abbreviations:pCR, pathological complete response; DFS, disease-free survival; OS, overallsurvival. [file 12885_2022_10109_MOESM3_ESM.pptx]

## Slide 1
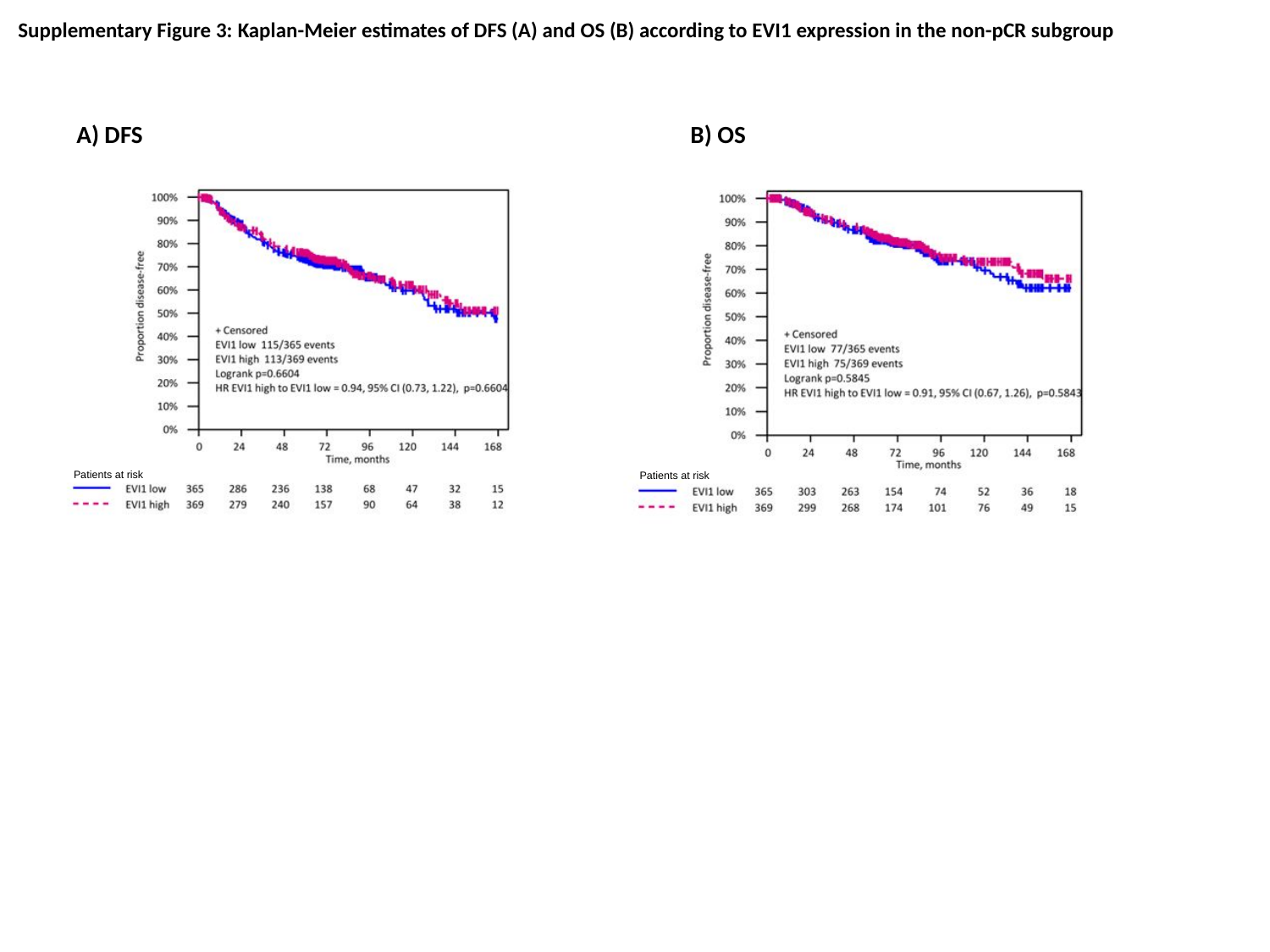

Supplementary Figure 3: Kaplan-Meier estimates of DFS (A) and OS (B) according to EVI1 expression in the non-pCR subgroup
A) DFS
B) OS
Patients at risk
Patients at risk
