# Supplementary material for: EVI1 expression in early-stage breast cancer patients treated with neoadjuvant chemotherapy
Source: BMC Cancer. 2022 Oct 5;22:1040. doi: 10.1186/s12885-022-10109-1 (PMC9533588; doi:10.1186/s12885-022-10109-1)
Supplement: Supplementary file 4 — Additional file 4: Supplementary Table 1. Correlation of baseline patient and tumor characteristics with EVI1 expression among BC subtypes. Supplementary Table 2. Multivariate analysis of EVI1 prognostic value in the entire cohort. Supplementary Table 3. pCR (ypT0 ypN0) rates overall and among breast cancer subtypes. Supplementary Table 4. Multivariate analysis of EVI1 prognostic value across BC subtypes. Supplementary Table 5. Univariate analysis of EVI1 prognostic value in patients with residual disease overall and among BC subtypes. [file 12885_2022_10109_MOESM4_ESM.docx]

# Supplementary Tables

## Supplementary Table 1: Correlation of baseline patient and tumor characteristics with EVI1 expression among BC subtypes

| **Subgroup/Parameter** | **Category** | **EVI1-Low (≤112.16)**  **N (%)** | **High (≥112.17)**  **N (%)** | **Overall (N=882)**  **N** | **p-value** |
| --- | --- | --- | --- | --- | --- |
| **HR+/HER2- (N=448)** |  |  |  |  |  |
| **Age, years** |  |  |  | **448** |  |
|  | ≤50 | 109 (47.0%) | 123 (53.0%) | 232 | 0.850 |
|  | >50 | 104 (48.1%) | 112 (51.9%) | 216 |  |
| **Tumor size** |  |  |  | **447** |  |
|  | cT1-2 | 140 (43.2%) | 184 (56.8%) | 324 | 0.004 |
|  | cT3-4a-d | 72 (58.5%) | 51 (41.5%) | 123 |  |
|  | missing | 1 | 0 | 1 |  |
| **Nodal status** |  |  |  | **439** |  |
|  | cN-negative | 99 (48.5%) | 105 (51.5%) | 204 | 0.924 |
|  | cN-positive | 112 (47.7%) | 123 (52.3%) | 235 |  |
|  | missing | 2 | 7 | 9 |  |
| **Tumor grade** |  |  |  | **436** |  |
|  | G1-2 | 162 (50.6%) | 158 (49.4%) | 320 | 0.066 |
|  | G3 | 47 (40.5%) | 69 (59.5%) | 116 |  |
|  | missing | 4 | 8 | 12 |  |
| **Histological type** |  |  |  | **448** |  |
|  | Ductal invasive | 194 (48.1% | 209 (51.9% | 403 | 0.530 |
|  | Non-ductal | 19 (42.2%) | 26 (57.8%) | 45 |  |
| **HR+/HER2+ (N=132)** |  |  |  |  |  |
| **Age, years** |  |  |  | **132** |  |
|  | ≤50 | 43 (55.8%) | 34 (44.2%) | 77 | 1.000 |
|  | >50 | 30 (54.5%) | 25 (45.5%) | 55 |  |
| **Tumor size** |  |  |  | **130** |  |
|  | cT1-2 | 45 (51.7% | 42 (48.3%) | 87 | 0.358 |
|  | cT3-4a-d | 26 (60.5%) | 17 (39.5%) | 43 |  |
|  | missing | 2 | 0 | 2 |  |
| **Nodal status** |  |  |  | **129** |  |
|  | cN-negative | 31 (52.5%) | 28 (47.5%) | 59 | 0.723 |
|  | cN-positive | 40 (57.1%) | 30 (42.9%) | 70 |  |
|  | missing | 2 | 1 | 3 |  |
| **Tumor grade** |  |  |  | 127 |  |
|  | G1-2 | 49 (59.0%) | 34 (41.0%) | 83 | 0.353 |
|  | G3 | 22 (50.0%) | 22 (50.0%) | 44 |  |
|  | missing | 2 | 3 | 5 |  |
| **Histological type** |  |  |  | 132 |  |
|  | Ductal invasive | 68 (54.4%) | 57 (45.6%) | 125 | 0.460 |
|  | Non-ductal | 5 (71.4%) | 2 (28.6%) | 7 |  |
| **HR-/HER2+ (N=86)** |  |  |  |  |  |
| **Age, years** |  |  |  | **86** |  |
|  | ≤50 | 24 (54.5%) | 20 (45.5%) | 44 | 0.291 |
|  | >50 | 18 (42.9%) | 24 (57.1%) | 42 |  |
| **Tumor size** |  |  |  | **86** |  |
|  | cT1-2 | 24 (46.2%) | 28 (53.8%) | 52 | 0.660 |
|  | cT3-4a-d | 18 (52.9%) | 16 (47.1%) | 34 |  |
| **Nodal status** |  |  |  | **86** |  |
|  | cN-negative | 15 (53.6%) | 13 (46.4%) | 28 | 0.647 |
|  | cN-positive | 27 (46.6%) | 31 (53.4%) | 58 |  |
| **Tumor grade** |  |  |  | **78** |  |
|  | G1-2 | 18 (50.0%) | 18 (50.0%) | 36 | 1.000 |
|  | G3 | 21 (50.0%) | 21 (50.0%) | 42 |  |
|  | missing | 3 | 5 | 8 |  |
| **Histological type** |  |  |  | 86 |  |
|  | Ductal invasive | 39 (50.6%) | 38 (49.4%) | 77 | 0.485 |
|  | Non-ductal | 3 (33.3%) | 6 (66.7%) | 9 |  |
| **TNBC (N=216)** |  |  |  |  |  |
| **Age, years** |  |  |  | **216** |  |
|  | ≤50 | 55 (44.4%) | 69 (55.6%) | 124 | 0.338 |
|  | >50 | 47 (51.1%) | 45 (48.9%) | 92 |  |
| **Tumor size** |  |  |  | **216** |  |
|  | cT1-2 | 65 (63.7%) | 83 (72.8%) | 148 | 0.187 |
|  | cT3-4a-d | 37 (36.3%) | 31 (27.2%) | 68 |  |
| **Nodal status** |  |  |  | **213** |  |
|  | cN-negative | 47 (45.6%) | 56 (54.4%) | 103 | 0.784 |
|  | cN-positive | 53 (48.2%) | 57 (51.8%) | 110 |  |
|  | missing | 2 | 1 | 3 |  |
| **Tumor grade** |  |  |  | **202** |  |
|  | G1-2 | 33 (42.9%) | 44 (57.1%) | 77 | 0.310 |
|  | G3 | 64 (51.2%) | 61 (48.8%) | 125 |  |
|  | missing | 5 | 9 | 14 |  |
| **Histological type** |  |  |  | **216** |  |
|  | Ductal invasive | 84 (45.2%) | 102 (54.8%) | 186 | 0.168 |
|  | Non-ductal | 18 (60.0%) | 12 (40.0%) | 30 |  |

Abbreviations: HR, hormone receptor; HER2, human epidermal growth factor receptor 2; TNBC, triple-negative breast cancer

## Supplementary Table 2: Multivariate analysis of EVI1 prognostic value in the entire cohort

| **Parameter** | **Category** | **pCR** | | **DFS** | | **OS** | |
| --- | --- | --- | --- | --- | --- | --- | --- |
|  |  | **Odds ratio (95%CI)** | **p-value** | **Hazard ratio (95%CI)** | **p-value** | **Hazard ratio (95%CI)** | **p-value** |
| **EVI1 continuous expression** | - | 1.13  (0.77-1.65) | 0.542 | 0.92  (0.72-1.17) | 0.488 | 0.85  (0.63-1.13) | 0.250 |
| age | >50 vs ≤ 50 | 0.53  (0.36-0.78) | 0.001 | 1.14  (0.90-1.45) | 0.287 | 1.14  (0.85-1.55) | 0.382 |
| cT | cT3-4 vs cT1-2 | 0.70  (0.46-1.08) | 0.110 | 1.84  (1.44-2.36) | <0.001 | 1.86  (1.36-2.53) | <0.001 |
| cN | cN+ vs cN- | 1.27  (0.87-1.86) | 0.212 | 1.84  (1.42-2.38) | <0.001 | 2.04  (1.46-2.85) | <0.001 |
| Tumor grade | G3 vs G1-2 | 2.52  (1.74-3.64) | <0.001 | 1.10  (0.85-1.40) | 0.502 | 1.23  (0.90-1.68) | 0.194 |
| Histological type | Non-ductal vs ductal invasive | 0.88  (0.46-1.70) | 0.707 | 1.52  (1.05-2.22) | 0.028 | 1.41  (0.87-2.27) | 0.160 |
| pCR | Yes vs no | - | - | 0.42  (0.27-0.66) | <0.001 | 0.27  (0.14-0.53) | <0.001 |
| **EVI1 dichotomized expression** | High vs low | 1.10  (0.76-1.60) | 0.606 | 0.96  (0.76-1.23) | 0.767 | 0.92  (0.68-1.25) | 0.606 |
| age | >50 vs ≤ 50 | 0.53  (0.36-0.78) | 0.001 | 1.14  (0.89-1.45) | 0.301 | 1.14  (0.84-1.54) | 0.406 |
| cT | cT3-4 vs cT1-2 | 0.70  (0.45-1.08) | 0.107 | 1.85  (1.44-2.37) | 0.000 | 1.88  (1.38-2.55) | <0.001 |
| cN | cN+ vs cN- | 1.27  (0.87-1.86) | 0.212 | 1.84  (1.42- 2.38) | 0.000 | 2.04  (1.46-2.84) | <0.001 |
| Grading | G3 vs G1-2 | 2.52  (1.74-3.65) | <0.001 | 1.09  (0.85-1.40) | 0.516 | 1.23  (0.90-1.68) | 0.194 |
| Histological type | Non-ductal vs ductal invasive | 0.89  (0.46-1.71) | 0.720 | 1.52  (1.04-2.21) | 0.029 | 1.40  (0.87-2.25) | 0.172 |
| pCR | Yes vs no | - | - | 0.42  (0.27-0.66) | <0.001 | 0.27  (0.14-0.53) | <0.001 |

Note, for regression analyses EVI1 continuous expression was transformed at units log10 increase;

CI, confidence interval; pCR, pathological complete response, DFS, disease-free survival, OS, overall survival

## Supplementary Table 3: pCR (ypT0 ypN0) rates overall and among breast cancer subtypes

| **Parameter** | **EVI1-low**  **N (%)** | **EVI1-high**  **N (%)** | **Overall**  **N** | **p-value** |
| --- | --- | --- | --- | --- |
| **Entire cohort** | **N=497** | **N=496** | **N=993** | 0.202 |
| No pCR | 422 (84.9%) | 406 (81.9%) | 828 |  |
| Yes pCR | 75 (15.1%) | 90 (18.1%) | 165 |  |
|  |  |  |  |  |
| **BC subtypes** |  |  | **N=882** |  |
| **HR+/HER2-** | **N=213** | **N=235** | **N=448** | 1.000 |
| No pCR | 196 (92.0%) | 216 (91.9%) | 412 |  |
| Yes pCR | 17 (8.0%) | 19 (8.1%) | 36 |  |
| **HR+/HER2+** | **N=73** | **N=59** | **N=132** | 1.000 |
| No pCR | 64 (87.7%) | 52 (88.1%) | 116 |  |
| Yes pCR | 9 (12.3%) | 7 (11.9%) | 16 |  |
| **HR-/HER2+** | **N=42** | **N=44** | **N=86** | 0.639 |
| No pCR | 31 (73.8%) | 30 (68.2%) | 61 |  |
| Yes pCR | 11 (26.2%) | 14 (31.8%) | 25 |  |
| **TNBC** | **N=102** | **N=114** | **N=216** | 0.114 |
| No pCR | 74 (72.5%) | 71 (62.3%) | 145 |  |
| Yes pCR | 28 (27.5%) | 43 (37.7%) | 71 |  |
| **Unknown** | N=67 | N=44 | **N=111** |  |
| No pCR | 57 (85.1%) | 37 (84.1%) | 94 |  |
| Yes pCR | 10 (14.9%) | 7 (15.9%) | 17 |  |

Note, pCR rates in the entire cohort and among breast cancer subtypes were estimated by 2-sided chi-square test.

## Supplementary Table 4: Multivariate analysis of EVI1 prognostic value across BC subtypes

| **BC subtype/parameter** | **Category** | **pCR** | | **DFS** | | **OS** | |
| --- | --- | --- | --- | --- | --- | --- | --- |
|  |  | **Odds ratio (95% CI)** | **p-value** | **Hazard ratio**  **(95% CI)** | **p-value** | **Hazard ratio**  **(95% CI)** | **p-value** |
| ***EVI1 continuous*** |  |  |  |  |  |  |  |
| **HR+/HER2-** |  |  |  |  |  |  |  |
| EVI1 | continuous | 0.98  (0.46-2.11) | 0.959 | 1.14  (0.74-1.76) | 0.562 | 0.96  (0.56-1.67) | 0.896 |
| age | >50 vs ≤50 | 0.41  (0.19-0.91) | 0.028 | 1.57  (1.06-2.34) | 0.025 | 2.45 (1.43-4.20) | 0.001 |
| cT | cT3-4 vs cT1-2 | 1.38  (0.60-3.16) | 0.445 | 1.73  (1.14-2.64) | 0.010 | 1.90  (1.12-3.23) | 0.018 |
| cN | cN+ vs cN- | 0.77  (0.36-1.64) | 0.492 | 2.05  (1.34-3.12) | 0.001 | 2.31  (1.31-4.08) | 0.004 |
| Grading | G3 vs G1-2 | 3.40  (1.61-7.19) | 0.001 | 0.87  (0.55-1.40) | 0.569 | 1.19  (0.63-2.00) | 0.705 |
| Histological type | Non-ductal vs ductal-invasive | 0.64  (0.14-2.88) | 0.564 | 1.22  (0.67-2.24) | 0.519 | 0.82  (0.33-2.06) | 0.669 |
| pCR | Yes vs no | - | - | 0.39  (0.12-1.26) | 0.115 | 0.47  (0.11-1.95) | 0.298 |
| **HR+/HER2+** |  |  |  |  |  |  |  |
| EVI1 | - | 0.91  (0.31-2.70) | 0.862 | 0.91  (0.52-1.61) | 0.754 | 0.84  (0.40-1.73) | 0.627 |
| age | >50 vs ≤50 | 1.25  (0.35-4.50) | 0.734 | 1.27  (0.67-2.45) | 0.461 | 0.76  (0.31-1.86) | 0.552 |
| cT | cT3-4 vs cT1-2 | 0.85  (0.23-3.11) | 0.802 | 1.84  (0.98-3.48) | 0.059 | 1.60  (0.69-3.71) | 0.278 |
| cN | cN+ vs cN- | 1.03  (0.29-3.68) | 0.961 | 1.15  (0.61-2.16) | 0.674 | 1.80  (0.76-4.18) | 0.172 |
| Grading | G3 vs G1-2 | 0.33  (0.07-1.61) | 0.170 | 0.81  (0.41-1.61) | 0.551 | 0.83  (0.34-2.02) | 0.683 |
| Histological type | Non-ductal vs ductal-invasive | 2.65  (0.25-28.51) | 0.422 | 2.66  (0.60-11.68) | 0.196 | 3.58  (0.79-16.36) | 0.099 |
| pCR | Yes vs no | - | - | 0.85  (0.26-2.80) | 0.788 | 0.60  (0.08-4.65) | 0.629 |
| **HR-/HER2+** |  |  |  |  |  |  |  |
| EVI1 | - | 1.00  (0.995-1.01) | 0.293 | 0.69  (0.33-1.45) | 0.328 | 0.63  (0.28-1.41) | 0.260 |
| age | >50 vs ≤50 | 0.54  (0.17-1.71) | 0.030 | 0.94 (0.43-2.08) | 0.883 | 0.85  (0.32-2.27) | 0.741 |
| cT | cT3-4 vs cT1-2 | 0.21  (0.05-0.86) | 0.836 | 0.72  (0.30-1.68) | 0.443 | 1.04  (0.36-3.04) | 0.944 |
| cN | cN+ vs cN- | 1.14  (0.33-3.90) | 0.729 | 3.01  (1.01-9.01) | 0.049 | 2.01  (0.57-7.15) | 0.281 |
| Grading | G3 vs G1-2 | 1.22  (0.39-3.84) | 0.999 | 0.75  (0.34-1.66) | 0.485 | 0.87  (0.33-2.30) | 0.773 |
| Histological type | Non-ductal vs ductal-invasive | <0.01  <0.01-n.a.) | 0.293 | 2.76  (0.67-11.28) | 0.158 | 3.48  (0.77-15.81) | 0.106 |
| pCR | Yes vs no | - | - | 0.35  (0.12-1.07) | 0.065 | 0.32  (0.07-1.50) | 0.149 |
| **TNBC** |  |  |  |  |  |  |  |
| EVI1 | - | 1.00  (0.999-1.01) | 0.128 | 0.82  (0.51-1.31) | 0.399 | 0.85  (0.50-1.46) | 0.556 |
| age | >50 vs ≤50 | 0.41  (0.21-0.80) | 0.009 | 0.81  (0.49-1.33) | 0.406 | 0.67  (0.37-1.19) | 0.169 |
| cT | cT3-4 vs cT1-2 | 0.45  (0.21-0.96) | 0.040 | 2.00  (1.21-3.29) | 0.007 | 1.93  (1.08-3.45) | 0.027 |
| cN | cN+ vs cN- | 2.05  (1.05-3.98) | 0.034 | 1.90  (1.13-3.18) | 0.015 | 1.96  (1.07-3.58) | 0.030 |
| Grading | G3 vs G1-2 | 2.08  (1.04-4.16) | 0.039 | 0.74  (0.45-1.20) | 0.224 | 0.66  (0.37-1.17) | 0.156 |
| Histological type | Non-ductal vs ductal-invasive | 1.36  (0.52-3.58) | 0.530 | 2.11  (1.09-4.06) | 0.026 | 1.68  (0.74-3.85) | 0.219 |
| pCR | Yes vs no | - | - | 0.24  (0.12-0.49) | <0.001 | 0.11  (0.03-0.35) | <0.001 |
| ***EVI1 dichotomized*** |  | **pCR** |  | **DFS** |  | **OS** |  |
| **HR+/HER2-** |  |  |  |  |  |  |  |
| EVI1 | High vs low | 0.76  (0.36-1.61) | 0.476 | 1.14  (0.76-1.70) | 0.529 | 1.16  (0.69-1.95) | 0.586 |
| age | >50 vs ≤50 | 0.41  (0.19-0.91) | 0.028 | 1.58  (1.06-2.35) | 0.024 | 2.46  (1.44-4.23) | 0.001 |
| cT | cT3-4 vs cT1-2 | 1.33  (0.58-3.04) | 0.502 | 1.74  (1.14-2.65) | 0.010 | 1.96  (1.15-3.33) | 0.014 |
| cN | cN+ vs cN- | 0.77  (0.36-1.65) | 0.498 | 2.05  (1.35-3.13) | 0.001 | 2.28  (1.29-4.00) | 0.004 |
| Grading | G3 vs G1-2 | 3.47  (1.64-7.33) | 0.001 | 0.87  (0.55-1.40) | 0.574 | 1.09  (0.61-1.94) | 0.766 |
| Histological type | Non-ductal vs ductal-invasive | 0.64  (0.14-2.87) | 0.561 | 1.20  (0.65-2.21) | 0.556 | 0.79  (0.31-2.00) | 0.623 |
| pCR | Yes vs no | - | - | 0.39  (0.12-1.26) | 0.117 | 0.48  (0.12-2.00) | 0.314 |
| **HR+/HER2+** |  |  |  |  |  |  |  |
| EVI1 | High vs low | 0.99  (0.29-3.42) | 0.987 | 1.17  (0.62-2.20) | 0.622 | 0.95  (0.40-2.28) | 0.914 |
| age | >50 vs ≤50 | 1.25  (0.35-4.49) | 0.736 | 1.30  (0.68-2.50) | 0.429 | 0.77  (0.31-1.87) | 0.559 |
| cT | cT3-4 vs cT1-2 | 0.84  (0.23-3.11) | 0.794 | 1.83  (0.98-3.44) | 0.059 | 1.55  (0.68-3.57) | 0.301 |
| cN | cN+ vs cN- | 1.04  (0.29-3.71) | 0.948 | 1.14  (0.61-2.15) | 0.678 | 1.79  (0.77-4.17) | 0.176 |
| Grading | G3 vs G1-2 | 0.33  (0.07-1.60) | 0.168 | 0.79  (0.39-1.58) | 0.500 | 0.82  (0.34-2.02) | 0.668 |
| Histological type | Non-ductal vs ductal-invasive | 2.64  (0.24-28.63) | 0.424 | 2.87  (0.64-12.85) | 0.169 | 3.64  (0.76-17.40) | 0.106 |
| pCR | Yes vs no | - | - | 0.88  (0.27-2.90) | 0.835 | 0.63  (0.08-4.82) | 0.655 |
| **HR-/HER2+** |  |  |  |  |  |  |  |
| EVI1 | High vs low | 1.38  (0.46-4.14) | 0.570 | 0.74  (0.34-1.62) | 0.444 | 0.52  (0.19-1.38) | 0.189 |
| age | >50 vs ≤50 | 0.55  (0.17-1.74) | 0.311 | 0.90  (0.41-1.95) | 0.785 | 0.78  (0.30-2.03) | 0.616 |
| cT | cT3-4 vs cT1-2 | 0.21  (0.05-0.83) | 0.027 | 0.77  (0.34-1.75) | 0.528 | 1.09  (0.39-3.08) | 0.871 |
| cN | cN+ vs cN- | 1.15  (0.34-3.93) | 0.828 | 3.03  (1.01-9.07) | 0.048 | 2.31  (0.64-8.39) | 0.204 |
| Grading | G3 vs G1-2 | 1.26  (0.41-3.93) | 0.688 | 0.78  (0.35-1.71) | 0.529 | 0.87  (0.33-2.33) | 0.786 |
| Histological type | Non-ductal vs ductal-invasive | <0.01 (<0.01-n.a.) | 0.999 | 2.64  (0.65-10.70) | 0.175 | 3.62  (0.78-16.73) | 0.100 |
| pCR | Yes vs no | - | - | 0.34  (0.11-1.05) | 0.061 | 0.30  (0.06-1.43) | 0.131 |
| **TNBC** |  |  |  |  |  |  |  |
| EVI1 | High vs low | 1.55  (0.81-2.96) | 0.188 | 1.04  (0.63-1.73) | 0.874 | 0.99  (0.55-1.78) | 0.980 |
| age | >50 vs ≤50 | 0.41  (0.21-0.80) | 0.009 | 0.80  (0.49-1.31) | 0.369 | 0.66  (0.37-1.17) | 0.153 |
| cT | cT3-4 vs cT1-2 | 0.44  (0.21-0.95) | 0.037 | 2.05  (1.24-3.39) | 0.005 | 1.97  (1.10-3.54) | 0.022 |
| cN | cN+ vs cN- | 2.02  (1.04-3.91) | 0.038 | 1.93  (1.15-3.23) | 0.012 | 1.99  (1.09-3.64) | 0.025 |
| Grading | G3 vs G1-2 | 2.15  (1.07-4.32) | 0.032 | 0.74 (0.45-1.21) | 0.233 | 0.67  (0.38-1.19) | 0.173 |
| Histological type | Non-ductal vs ductal-invasive | 1.43  (0.54-3.77) | 0.471 | 2.14  (1.10-4.19) | 0.026 | 1.68  (0.72-3.89) | 0.228 |
| pCR | Yes vs no | - | - | 0.23  (0.11-0.48) | <0.001 | 0.10  (0.03-0.34) | <0.001 |

Note, for regression analyses EVI1 continuous expression was transformed at units log10 increase;

CI, confidence interval; pCR, pathological complete response, DFS, disease-free survival, OS, overall survival; HR, hormone receptor; HER2, human epidermal growth factor receptor 2; TNBC, triple-negative breast cancer

## Supplementary Table 5: Univariate analysis of EVI1 prognostic value in patients with residual disease overall and among BC subtypes

| **BC subtype** | **Category** | **DFS** | | **OS** | |
| --- | --- | --- | --- | --- | --- |
|  |  | **Hazard ratio (95% CI)** | **log-rank p-value** | **Hazard ratio (95% CI)** | **log-rank p-value** |
| **overall** | EVI1 high vs low | 0.94  (0.73-1.22) | 0.660 | 0.91  (0.67-1.26) | 0.585 |
| **HR+/HER2-** | EVI1 high vs low | 1.07  (0.73-1.58) | 0.731 | 1.03  (0.63-1.70) | 0.895 |
| **HR+/HER2+** | EVI1 high vs low | 1.07  (0.57-2.00) | 0.833 | 0.92  (0.41-2.07) | 0.833 |
| **HR-/HER2+** | EVI1 high vs low | 0.74  (0.34-1.64) | 0.456 | 0.55  (0.21-1.49) | 0.234 |
| **TNBC** | EVI1 high vs low | 0.85  (0.51-1.42) | 0.537 | 0.93  (0.53-1.63) | 0.796 |

CI, confidence interval; DFS, disease-free survival, OS, overall survival; HR, hormone receptor; HER2, human epidermal growth factor receptor 2; TNBC, triple-negative breast cancer
